# Effect of risk messages on risk appraisals, attitudes, ambivalence, and willingness to smoke hookah in young adults

**DOI:** 10.1080/21642850.2020.1730844

**Published:** 2020-02-20

**Authors:** Darren Mays, Andrea C. Johnson, Lilianna Phan, Kenneth P. Tercyak, Kathryn Rehberg, Isaac Lipkus

**Affiliations:** a Lombardi Comprehensive Cancer Center, Georgetown University Medical Center, Washington, DC, USA; b Department of Prevention & Community Health, Milken Institute School of Public Health, George Washington University, Washington, DC, USA; c Duke University School of Nursing, Durham, NC, USA

**Keywords:** Young adults, tobacco, risk communication, attitude, ambivalence

## Abstract

**Objective:** We examined effects of hookah tobacco risk messages on risk appraisals, attitudes towards hookah, ambivalence about hookah use, and willingness to smoke in young adults aged 18–30 years (*n *= 234).

**Design:** In an online experiment, participants completed pre-exposure measures and were randomized to hookah tobacco risk messages or to a no message control condition.

**Main Outcome Measures:** Risk appraisals, attitudes, ambivalence, and willingness to smoke hookah.

**Results:** Those who viewed risk messages reported greater risk appraisals (M 4.50, SD 1.17 vs. M 3.87, SD 1.16, *p* < .001), less positive attitudes (M −0.56, SD 1.24, vs. M 0.39, SD 1.35, *p* < .001), greater ambivalence (M 3.86, SD 1.26, vs. M 3.08, SD 1.32, *p* < .001), and less willingness to smoke than controls (M 4.48, SD 1.27, vs. M 4.85, SD 1.37, *p* = .034). Structural equation modeling demonstrated messages reduced willingness to smoke by evoking less positive attitudes (*b* = −0.15, 95% CI −0.32, −0.05) and by the effect of heightened risk appraisals on less positive attitudes (*b* = −0.14, 95% CI −0.30, −0.07).

**Conclusions:** Honing messages and understanding their mechanisms of action are necessary to produce more effective interventions to address hookah and other tobacco use in young adults.

## Introduction

Messages communicating the risks of behaviors that are harmful to health (e.g. tobacco use) are an important component of public health interventions to prevent and reduce the disease burden associated with such behaviors (e.g. cancer). Often, these messages target young people who are at a developmental stage when risk behaviors emerge, their frequency and/or intensity accelerates, and habits solidify (Villanti, Niaura, Abrams, & Mermelstein, 2019). Periods such as young adulthood present a critical window of opportunity when interventions such as risk messages are needed to promote healthy behavior (Villanti et al., 2019). To achieve optimal impact on behavioral outcomes, it is critical to understand the cognitive and affective constructs through which risk messages operate to influence behavior.

Risk appraisals are important theoretical targets of messages communicating risks to promote behavior change among young people (Sheeran, Harris, & Epton, 2014). Evidence demonstrates that change in risk appraisals in response to such interventions is associated with change in target behavioral outcomes (Sheeran et al., 2014). However, to maximize their potential effects, interventions should target other important psychological constructs that predict behavior change, such as attitudes toward a behavior (i.e. one’s global evaluations of a target behavior) (Ajzen, 2001; Fishbein & Ajzen, 2010). A recent meta-analysis lends empirical support to this idea, demonstrating that measures of behavioral attitudes are strongly correlated with behavioral intention and behavior (McEachan et al., 2016). Other theoretical work, such as the affect heuristic, highlights the interplay between attitudinal measures (e.g. good/bad, pleasant/unpleasant) and risk appraisals indicating higher risk appraisals should be related to negative attitudes towards a target risk behavior (Slovic, Finucane, Peters, & MacGregor, 2004).

Another line of research has examined ambivalence, or the extent to which young people feel conflict about risk behaviors like tobacco use, as a psychological construct that risk messages can affect to motivate behavior change (Hohman, Crano, & Niedbala, 2016). For example, young adults who are ambivalent about smoking cigarettes have greater desire to quit (Lipkus, Green, Feaganes, & Sedikides, 2001) and are more likely to seek out information about tobacco (Zhao & Cai, 2008). Interventions that enhance feelings of ambivalence about risk behaviors may spur young people to avoid risky behavior in order to reduce the ambivalence they feel (Lipkus & Noonan, 2017). Messages conveying risks of a target behavior may increase ambivalence among young people by affecting their beliefs about the associated risks (i.e. increasing risk appraisals) against a background of other positive beliefs (e.g. smoking is a fun social event).

Hookah (i.e. waterpipe, shisha) tobacco use among young adults serves as an important example for understanding the effects of risk messages on constructs such as risk appraisals, behavioral attitudes, and ambivalence for several reasons. First, according to recent population data about 30% of U.S. young adult non-users are susceptible to initiating hookah (Mays et al., 2016a) and about 11% of U.S. young adults are current (i.e. within the past month) hookah smokers (Kasza et al., 2017). Hookah tobacco smoking exposes users to harmful toxicants (e.g. carcinogens, heavy metals) similar to those in cigarette smoke (Shihadeh et al., 2015), it is associated with negative short- and long-term health effects (El-Zaatari, Chami, & Zaatari, 2015; Montazeri, Nyiraneza, El-Katerji, & Little, 2017), and the nicotine exposure is sufficient to produce addiction (Aboaziza & Eissenberg, 2015; Bahelah et al., 2016; Sidani, Shensa, Shiffman, Switzer, & Primack, 2016). Hookah tobacco smoking in young people is also associated with an increased risk of using other tobacco products in the future, such as cigarette smoking (Primack et al., 2015; Shepardson & Hustad, 2016). Taken together, this evidence indicates young adults’ hookah tobacco use warrants intervention.

Second, young adults’ hookah tobacco use is influenced by multiple psychological factors that risk messages may affect to produce behavior change (Akl et al., 2015). Young adults do not view hookah tobacco as harmful or addictive, beliefs that contribute to the high prevalence of use in young adults (Castaneda, Barnett, Soule, & Young, 2016; Hair et al., 2017; Lipkus & Mays, 2018; Mermelstein, 2014; Roberts, Klein, Berman, Berhane, & Ferketich, 2017). Young adults hold positive attitudes towards hookah tobacco that are associated with hookah use (Akl et al., 2015) and young adult non-users susceptible to hookah use (i.e. at risk of initiating) endorse more positive attitudes than those who are not susceptible (Lipkus, Mays, & Tercyak, 2017). With respect to ambivalence, whereas young adult hookah smokers express little ambivalence about hookah smoking, those who feel more conflicted report stronger risk appraisals, stronger desire to quit, and want more information about risks of hookah tobacco smoking (Lipkus & Noonan, 2017).

Finally, research on behavioral interventions targeting hookah tobacco use among young adults is extremely limited and gaps in the existing research warrant investigation (Lopez, Eissenberg, Jaafar, & Afifi, 2017). Some studies have examined effects of hookah tobacco risk messages on measures of risk appraisals and behavioral motivation. For example, Lipkus and colleagues (2011) tested effects of education messages about risks of hookah tobacco in college hookah smokers. Messages increased risk appraisals and desire to quit (Lipkus, Eissenberg, Schwartz-Bloom, Prokhorov, & Levy, 2011). Mays and colleagues (2016) examined effects of messages conveying health harms only and messages communicating both health harms and addictiveness of hookah tobacco on similar outcomes (Mays, Tercyak, & Lipkus, 2016). The messages increased risk appraisals and produced greater desire to quit in young adult hookah smokers. Prior research also indicates messages communicating the risks of hookah tobacco use produce less positive attitudes towards hookah tobacco and increase ambivalence among young adults who are susceptible non-users (Lipkus et al., 2017). However, among young adult hookah smokers there has not been research to examine if attitudes towards hookah tobacco or ambivalence shift in response to risk messages.

In this experimental study, we build from prior research investigating effects of hookah tobacco risk messages among young adult hookah tobacco smokers in two important ways. First, in addition to risk appraisals our objective was to examine message effects on outcomes not assessed in prior studies with young adult hookah smokers, including attitudes towards hookah tobacco and ambivalence about hookah tobacco. We hypothesized that hookah tobacco risk messages have a significant effect on these outcomes when comparing those exposed to the messages to those not exposed to messages. Second, we examined if risk appraisals, attitudes towards hookah tobacco, and ambivalence about hookah tobacco mediate message effects on willingness to smoke hookah to better understand potential mechanisms of risk message effects.

## Methods

### Setting

Study participants were recruited in August 2016 through the crowdsourcing website Amazon Mechanical Turk for an online experiment. Mechanical Turk has been used in similar studies testing the effects of tobacco messages (Kowitt et al., 2019; Lipkus et al., 2017; Mays et al., 2016b; Pearson et al., 2016; Seidenberg, Jo, & Ribisl, 2018) and research demonstrates its validity for this purpose (Jeong et al., 2018; Kraemer, Strasser, Lindblom, Niaura, & Mays, 2017). Although the online recruitment generates a convenience sample, recent evidence demonstrates correlational and experimental studies conducted through Mechanical Turk for tobacco research produce comparable effects to those using population-based samples (Jeong et al., 2018; Kraemer et al., 2017). Data quality assurance measures included prohibiting duplicate responses and using verification to prevent automated completion (i.e. by bots). All study procedures occurred online at a single timepoint. Potential participants read a brief study description, and those who were interested proceeded to eligibility screening questions.

### Participants

Eligible participants were young adults aged 18–30 years who self-reported smoking hookah tobacco at least once in the past month (Mays et al., 2016b). Participation was also restricted to those with Mechanical Turk accounts registered in the U.S. Our goal was to recruit at least 100 participants in each experimental condition to achieve adequate statistical power to detect mean differences in outcomes comparable to similar, previous studies (Lipkus et al., 2017; Mays et al., 2016b). Those meeting eligibility criteria proceeded to a complete study description and an online informed consent form to complete enrollment.

### Study design

Eligible, consenting participants completed initial measures of demographic characteristics, history of hookah use, cigarette smoking, and other tobacco product use. Participants were then randomized in approximately equal numbers to one of two conditions. Participants randomized to the control condition proceeded to complete study measures only. Participants randomized to the risk messaging condition viewed information conveying health harms and addictiveness of hookah tobacco use presented as a series of six screens that participants reviewed at their own pace. Messages were theoretically-informed, based on evidence of health harms and addictiveness of hookah tobacco use, and relayed risks through text and visual imagery. Message content conveyed long term health risks (e.g. cancer, heart disease), short-term risks (e.g. infections from sharing a waterpipe), toxicant exposure from hookah smoke, and information on addictiveness. Previously, messages were pretested for content and acceptability and they were experimentally tested in independent samples of young adult hookah tobacco smokers and susceptible non-smokers (Lipkus et al., 2017; Mays et al., 2016b).

### Measures

*Demographics.* Demographic characteristics assessed included age, sex, race, ethnicity, educational attainment, and employment status.

*Hookah Tobacco Smoking.* Frequency of hookah tobacco smoking was measured with a single valid item asking participants if they smoked hookah tobacco daily, weekly, or monthly (Lipkus et al., 2011; Mays et al., 2016b).

*Cigarette Smoking.* Cigarette smoking was measured with two valid items to define current smokers as those who smoked 100 or more lifetime cigarettes and currently smoked cigarettes every day or some days (Hu et al., 2016). For descriptive purposes, we report the proportion of current cigarette smokers and current non-smokers.

*Other Tobacco Use.* To characterize other tobacco use in the sample, we measured if participants used electronic cigarettes, large cigars, little cigars/cigarillos, and/or smokeless tobacco in the past 30 days. For descriptive purposes, we report the proportion of those individuals using any other tobacco product in the past 30 days versus not.

*Risk Appraisals.* We used 4 items to capture participants’ hookah tobacco risk appraisals (i.e. perceived risks, worry about risks) (Lipkus & Mays, 2018). Perceived risk of harm was measured by asking ‘What do you think is your chance of getting a serious smoking-related disease, such as cancer, lung disease, or heart disease, if you were to continue smoking waterpipe tobacco?’ (1 = no chance to 7 = certain to happen). Worry about harm was measured by asking ‘How worried would you be about getting a serious smoking-related disease, such as cancer, lung disease, or heart disease, if you continue smoking waterpipe tobacco?’ (1 = not at all to 7 = very). Perceived risk of addiction was measured as ‘What do you think is your chance of becoming addicted to nicotine in waterpipe tobacco if you were to continue to smoke it?’ (1 = no chance to 7 = certain to happen). Finally, worry about addiction was assessed by asking ‘How worried would you be about becoming addicted to nicotine in waterpipe tobacco if you continue to smoke it?’ (1 = not at all to 7 = very). The 4 items had good internal consistency (α = 0.81) and they were averaged to create an overall risk appraisals score with higher values indicating stronger risk appraisals. Risk appraisal items were examined as a latent variable in mediation analyses.

*Attitudes.* We measured attitudes towards hookah tobacco using 4 valid items with bipolar response options ranging from −3 to 3 (Lipkus et al., 2017). Participants indicated whether they believed hookah tobacco was negative/positive, bad/good, dislike/like, unpleasant/pleasant. For analyses of observed variables, items were summed and averaged (α = 0.93) to create a score where higher values indicate more positive global attitudes. Attitude items were also examined as a latent variable in mediation analyses.

*Ambivalence.* We measured felt ambivalence toward hookah tobacco using 3 items (Lipkus & Noonan, 2017): mixed feelings, conflicted thoughts, and felt torn about hookah tobacco (1 = strongly disagree to 5 = strongly agree). Items had good internal consistency (α = 0.94) and responses were averaged to create a score with higher values indicating greater ambivalence. Ambivalence items were examined as a manifest variable in mediation analyses.

*Willingness to Smoke Hookah Tobacco.* Willingness to smoke hookah tobacco in the future was measured using 4 items (Lipkus & Mays, 2018). The items captured how likely participants would be to smoke hookah tobacco again in the future if offered it by a friend, how tempted they are to smoke hookah tobacco in the next year, if they saw themselves smoking hookah tobacco in the next year, and how curious they were about smoking hookah tobacco. Response options ranged from 1 = not at all/definitely not to 5 = very/definitely yes. The 4 items had good internal consistency (α = 0.84) and responses were averaged to create a score with higher values indicating greater willingness to smoke hookah. The 4 items capturing willingness to smoke hookah in the future were examined as a latent variable in mediation analyses.

### Statistical methods

We used descriptive statistics to characterize the study sample and bivariate tests (i.e. chi-square tests, *t*-tests) to assess for differences by study condition. No participant characteristics differed significantly by study condition so we did not adjust analyses for covariates. We characterized associations among risk appraisals, attitudes, ambivalence, and willingness to smoke hookah by examining Pearson’s correlations among them. Finally, we tested for differences in means for risk appraisals, attitudes, ambivalence, and willingness to smoke hookah by the study conditions using independent sample *t-*tests.

We used structural equation modeling (SEM) to explore if risk appraisals, attitudes, and ambivalence mediate message effects on willingness to smoke hookah. First, we specified the measurement model with latent variables for risk appraisals, attitudes, ambivalence, and willingness to smoke hookah. We examined the measurement model in a systematic series of steps, first assessing fit indices for each latent variable specified separately and once adequate fit was established for latent constructs individually examining fit of the complete measurement model with all variables. We identified the best fitting model at each of these steps by evaluating a combination of fit indices including Root Mean Square Error of Approximation (RMSEA), Comparative Fit Index (CFI), and Standardized Root Mean Square Residual (SRMR). We used the following values as benchmarks for assessing model fit: RMSEA < = 0.05 and 90% Confidence Interval 0.00-0.08; CFI >0.95, and SRMR < = 0.08 (Brown, 2015; Geiser, 2013; Hu & Bentler, 1999). In the measurement models for each latent variable and when latent variables were combined into a single measurement model, we examined modification indices to identify if model fit could be improved in ways that were logically and theoretically consistent with the model.

Once we achieved a measurement model that fit the data well, we estimated the direct and indirect (i.e. mediation) effects of the message exposure on willingness to smoke hookah in a structural model. Study condition was entered as a manifest variable where the risk messaging condition and was coded as 1 and the control condition was coded as 0. The structural model was based on guiding theoretical models characterizing the potential mediating roles of risk appraisals, attitudes, and ambivalence following exposure to study messages (Ajzen, 2001; Fishbein & Ajzen, 2010; Hohman et al., 2016; Lipkus & Noonan, 2017; Slovic et al., 2004). Specifically, we tested if risk appraisals were associated with attitudes, ambivalence, and willingness to smoke hookah and modeled the indirect effects of messages on willingness to smoke hookah tobacco through risk appraisals, attitudes, and ambivalence. We used full information maximum likelihood estimation and to estimate indirect effects a bias-corrected bootstrapping approach with 1000 resamples to address non-normality in the product of coefficients (Williams & Mackinnon, 2008). Asymmetric 95% confidence intervals (CIs) around estimates that do not include zero indicate statistically significant indirect effects. We used Mplus version 7.4 for structural equation modeling and SAS version 9.4 for all other analyses.

## Results

### Participants

In total 698 potentially interested individuals were screened for eligibility and 246 (35.2%) met study eligibility criteria. All eligible participants completed study procedures and were included in analyses. Sample characteristics are shown in Table 1. Overall, the sample was primarily male (66%), white (79%), had a college education or higher (87%), and smoked hookah monthly (52%) or weekly (37%) (Table 1). The average time to complete procedures was 9.9 min (SD 10.9, Median 7.3 min); as expected, time to complete procedures was greater among those randomized to the risk messaging condition (*M = *11.9, *SD *= 12.2) versus the control condition (*M* 8.2, *SD* 9.3, *p *= .007).

**Table 1.** Sample characteristics (*N* = 246).

	Mean (SD)	*n* (%)
Sex		
Female		83 (33.88%)
Male		162 (66.12%)
Age	25.48 (2.85)	
Race		
White		193 (78.78%)
Non-white		52 (21.22%)
Ethnicity		
Hispanic		35 (14.29%)
Non-Hispanic		210 (85.71%)
Education		
College Education and Higher		214 (86.99%)
Less Than College Education		32 (13.01%)
Employment		
Full Time Employed		161 (65.98%)
Not Full Time Employed		83 (34.02%)
Hookah Tobacco Smoking		
Monthly		127 (51.84%)
Weekly		91 (37.14%)
Daily		27 (11.02%)
Cigarette Smoking Status		
Current Smoker		133 (56.84%)
Non-Smoker		101 (43.16%)
Other Tobacco Use in Past 30 Days		
Yes		94 (38.21%)
No		152 (61.79%)

Note. SD* *= Standard Deviation. Some Ns for categories within variables do not sum to total sample size due to sporadic missing data (<5% of cases for any individual variable).

### Correlations among outcome variables

Bivariate correlations among hookah tobacco risk appraisals, attitudes, ambivalence, and willingness to smoke are displayed in Table 2. Lower risk appraisals (*r* = −0.22, *p* < .001) and more positive attitudes (*r* = 0.49, *p* < .001) were significantly correlated with willingness to smoke hookah. Higher risk appraisals were significantly correlated with less positive attitudes (*r* = −0.53, *p* < .001) and greater ambivalence (*r* = 0.44, *p* < .001). Additionally, greater ambivalence was correlated with less positive attitudes (*r* = −0.33, *p* < .001).

**Table 2.** Descriptive statistics and correlations for outcome variables.

	*M* (SD)	Correlations			
		1	2	3	4
1. Risk appraisals	4.2 (1.2)	1.00			
2. Attitudes	−0.06 (1.4)	−0.53***	1.00		
3. Ambivalence	3.5 (1.3)	0.44***	−0.33***	1.00	
4. Willingness to smoke Hookah	4.7 (1.3)	−0.22***	0.49***	−0.08	1.00

Note: Mean (Standard Deviation) displayed in the first column. Ranges are: risk appraisals 1–7, attitudes −3–3, ambivalence 1–5, and willingness to smoke Hookah 1–5. *p < .05; **p < .01; ***p < .001.

### Message effects

The tests of mean differences for outcome variables between the risk messaging and control conditions are shown in Table 3. Compared with participants in the control condition, participants viewing risk messages reported significantly greater risk appraisals (*M* 4.50, *SD* 1.17, *M* 3.87, *SD* 1.16, *p* < .001), less positive attitudes towards hookah (*M* −0.56, *SD* 1.24, *M* 0.39, *SD* 1.35, *p* < .001), greater ambivalence (*M* 3.86, *SD* 1.26, *M* 3.08, *SD* 1.32, *p *< .001), and less willingness to smoke hookah in the future (*M* 4.48, *SD* 1.27, *M* 4.85, *SD* 1.37, *p *=* *.034).

**Table 3.** Effect of risk messages on risk appraisals, attitudes, ambivalence, and willingness to smoke.

	Risk Messaging Condition	Control Condition	
Variable	*M* (SD)	*M* (SD)	*p*
Risk appraisals	4.50 (1.17)	3.87 (1.16)	<.001
Attitudes	−0.56 (1.24)	0.39 (1.35)	<.001
Ambivalence	3.86 (1.26)	3.08 (1.32)	<.001
Willingness to smoke Hookah	4.48 (1.27)	4.85 (1.37)	0.034

Note: Mean (Standard Deviation) displayed. Average ranges include: Risk Appraisals 1–7, Attitudes −3–3, Ambivalence 1–5, and Willingness to Smoke Hookah 1–5.

### Mediation analyses

*Measurement Model.* For the measurement model, evaluation of model fit for latent constructs and modification indices led us to correlate indicator items for latent variables to improve model fit. This included correlating items for risk appraisals (chance of disease, chance of becoming addicted), attitudes (dislike/like with negative/positive, dislike/like with bad/good), and willingness to smoke hookah (tempted to smoke and smoking in the next year with smoking if offered by a friend). For ambivalence, the 3 indicator variables loaded nearly identically with limited variance and suboptimal fit. As a result, we modeled ambivalence as a manifest variable using average scores as described above. Model fit for the latent variables individually after these modifications were as follows: risk appraisals (RMSEA = 0.06 [90% CI 0.00, 0.19], CFI = 1.00, SRMR = 0.001), attitudes (RMSEA <0.01 [90% CI 0.00, 0.12]; CFI = 1.00, SRMR = 0.001), willingness to smoke hookah (RMSEA <0.01 [90% CI 0.00, 0.11], CFI = 1.00, SRMR = 0.002).

**Figure 1.**
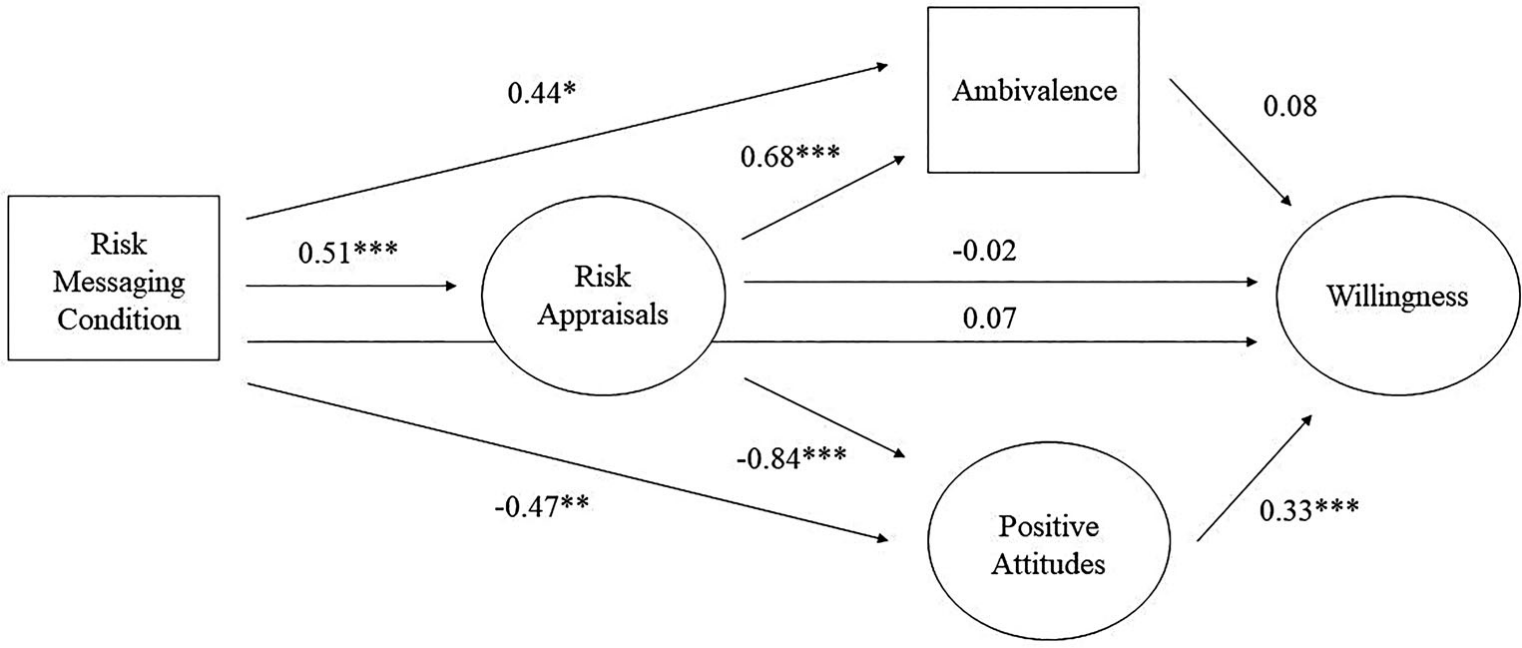
Final structural equation model. Note: Direct paths from the model illustrated here. Measurement model and factor loadings are not shown. Model fit statistics were RMSEA = 0.06 [90% CI = 0.03, 0.07], CFI = 0.97, SRMR = 0.06. *p<.05, **p<.01, ***p<.001.

*Structural Equation Model.* The final model had good fit (RMSEA = 0.06 [90% CI = 0.03, 0.07], CFI = 0.97, SRMR = 0.06). Figure 1 displays the direct paths in the model, and the results of the direct and indirect paths modeled are shown in Table 4. In this model the direct path of risk messages on willingness to smoke hookah was no longer significant, and there were two significant indirect paths with bootstrapped 95% confidence intervals that did not include zero. Risk messages affected willingness to smoke hookah tobacco by generating less positive attitudes (*b* = −0.15, 95% CI = −0.32, −0.05). Additionally, risk messages affected willingness to smoke hookah tobacco by heightening risk appraisals, which generated less positive attitudes (*b* = −0.14, 95% CI = −0.30, −0.06).

**Table 4.** Direct and indirect effect of risk messages on willingness to smoke hookah.

	*b* (95% CI)
Risk messaging condition > risk appraisals	0.51 (0.27, 0.76)
Risk messaging condition > attitudes	−0.47 (−0.79, −0.14)
Risk messaging condition > ambivalence	0.44 (0.10, 0.76)
Risk messaging condition > willingness	0.07 (−0.13, 0.29)
Risk appraisals > attitudes	−0.84 (−1.12, −0.62)
Risk appraisals > ambivalence	0.68 (0.42, 0.97)
Risk appraisals > willingness	−0.02 (−0.21, 0.15)
Attitudes > willingness	0.33 (0.18, 0.52)
Ambivalence > willingness	0.08 (−0.01, 0.20)
Risk messaging condition > risk appraisals > willingness	−0.01 (−0.11, 0.08)
Risk messaging condition > attitudes > willingness	−0.15 (−0.32, −0.05)
Risk messaging condition > ambivalence > willingness	0.04 (0.00, 0.12)
Risk messaging condition > risk appraisals > attitudes > willingness	−0.14 (−0.30, −0.06)
Risk messaging condition > risk appraisals > ambivalence > willingness	0.03 (0.00, 0.07)

Note: Unstandardized regression coefficients and 95% confidence intervals (CI) are displayed. 95% CIs that do not include 0 are statistically significant at *p* < .05.

## Discussion

Prior research demonstrates that messages communicating the risks of hookah tobacco use can affect risk appraisals and desire to quit in young adult hookah tobacco smokers (Lipkus et al., 2011; Mays et al., 2016b). Our study supports and extends these findings by showing that hookah tobacco risk messages affect risk appraisals as well as hookah tobacco attitudes and ambivalence toward hookah use. Although our mediation analysis should be considered preliminary given study limitations, findings highlight constructs that may play a key role in risk messages’ impact on hookah use. Message effects on willingness to smoke hookah tobacco were mediated by producing less positive attitudes towards hookah and by the effect of less positive attitudes by way of heightened risk appraisals. Results of this study advance the research on interventions for young adults’ hookah tobacco use and highlight research directions that are important in future studies.

Interventions that influence risk appraisals can change behavioral outcomes (Sheeran et al., 2014). There is compelling evidence that hookah tobacco use among young adults is driven in part by beliefs that it is safe (Akl et al., 2015). This supports the use of risk messages to address patterns of beliefs contributing to this behavior. Our findings show that the messages we designed on risks of health harm and addiction accomplished the intended goal of affecting young adult hookah smokers’ risk appraisals. This result is in line with similar studies in young adult hookah smokers (Lipkus et al., 2011; Mays et al., 2016b) and susceptible non-smokers (Lipkus et al., 2017). Currently, however, studies in this area including the present investigation are limited to cross-sectional examinations of message effects on outcomes such as risk appraisals. Prospective studies examining if the change in hookah tobacco risk appraisals in response to messages translates to behavior change are needed.

Consistent with behavioral models (Ajzen, 2001; Fishbein & Ajzen, 2010), our intervention shifted young adults’ attitudes towards hookah in a way that may motivate behavior change. Moreover, our mediation analysis highlights that attitudes may be an important mechanism through which risk messaging affects hookah use behavior. Attitudes explained message effects on willingness to smoke through two indirect paths, from messages to attitudes and from messages to risk appraisals to attitudes. These results align with theoretical models highlighting the important role of affective responses to risk information in behavioral decisions (Slovic et al., 2004). The findings are also consistent with the idea that cognitions about risk and affective attitudinal measures correlate with one another (Slovic et al., 2004). These findings are especially promising given the role that attitudes play in shaping behavior (Ajzen, 2001; Fishbein & Ajzen, 2010) and motivating behavior change (McEachan et al., 2016). This new addition to the research on hookah tobacco interventions warrants further study, particularly studying the effects of risk messages on hookah tobacco risk appraisals and attitudes over time, and considering developing message content that may more explicitly target behavioral attitudes in addition to risk appraisals.

We also drew from a relatively smaller body of research on the role of ambivalence in young adult hookah smokers (Lipkus & Noonan, 2017). Our study is among the first to demonstrate that risk communication messages can heighten ambivalence in young adult hookah tobacco smokers. This is an important result given that heightened ambivalence may motivate young hookah smokers to avoid hookah tobacco smoking in order to reduce the conflict they feel between their beliefs and their behavior (Lipkus & Noonan, 2017). In young adult hookah smokers, ambivalence is a construct where there is ample room to foster change, and interventions producing change in ambivalence may act to motivate cessation, seeking additional information about hookah tobacco use, or pursuing more intensive intervention (Lipkus & Noonan, 2017).

Although we found a direct effect of the messages on ambivalence, ambivalence was not a significant mediator of message effects on willingness to smoke. This differs from prior research on young adults who do not smoke hookah tobacco but are susceptible, which has identified ambivalence as a potential mediator of risk communication message effects (Lipkus et al., 2017). Notably, owing to the model fit we used a manifest variable for ambivalence in mediation analyses, which may have increased measurement error and affected results. Other factors that we did not assess may also be important to characterize the effect of heightened ambivalence on outcomes related to hookah tobacco use behavior. For example, one prior study showed that for messages conveying the risks of cigarette smoking, the effect of content designed to heighten ambivalence about smoking was moderated by the inclusion of information targeting social norms (Hohman et al., 2016). For many young adults, hookah tobacco smoking is a social behavior that often occurs in cafes, lounges, and other social contexts. It is possible that risk message effects on ambivalence vary based on factors such as young adults’ preexisting normative beliefs and usual contexts for hookah use. It may also be that message content specifically addressing beliefs about social aspects of hookah smoking (e.g. that smoking in settings such as cafes and lounges has associated risks) has a stronger effect on ambivalence. These are important questions to pursue in future studies to gain a more complete understanding of how risk messages affect ambivalence and if heightened ambivalence may motivate behavior change in young adult hookah smokers.

Our experimental design is a study strength, but our findings should be interpreted in light of important study limitations. The study included a convenience sample of young adult hookah smokers and generalizability may be limited. Despite this limitation, the demographic characteristics of the sample resemble those from population-based data on young adult hookah smokers in the U.S. (Robinson, Wang, Jackson, Donaldson, & Ryant, 2018). We relied on self-report measures collected at a single time point. The mediation analyses should be interpreted as preliminary because we did not examine prospective associations. Although informative, prospective data are needed to verify, replicate, and build from the findings observed here.

Despite these limitations, the findings of this study reinforce the important role that risk communication messages can play as an intervention to prevent and reduce young adults’ hookah tobacco use. We demonstrate message effects on risk appraisals, as well as constructs not yet studied in young adult hookah smokers in response to such messages including attitudes, ambivalence, and willingness to smoke hookah tobacco in the future. Future studies can build from this work by assessing prospective effects of risk messages over time and considering ways to formulate new message content to address other important beliefs driving young adults’ hookah tobacco use.
